# Yeast as a Heterologous Model System to Uncover Type III Effector Function

**DOI:** 10.1371/journal.ppat.1005360

**Published:** 2016-02-25

**Authors:** Crina Popa, Núria S. Coll, Marc Valls, Guido Sessa

**Affiliations:** 1 Genetics Department, Universitat de Barcelona, Barcelona, Catalonia, Spain; 2 Centre for Research in Agricultural Genomics (CSIC-IRTA-UAB-UB), Bellaterra, Catalonia, Spain; 3 Department of Molecular Biology and Ecology of Plants, Tel Aviv University, Tel Aviv, Israel; Stony Brook University, UNITED STATES

## Abstract

Type III effectors (T3E) are key virulence proteins that are injected by bacterial pathogens inside the cells of their host to subvert cellular processes and contribute to disease. The budding yeast *Saccharomyces cerevisiae* represents an important heterologous system for the functional characterisation of T3E proteins in a eukaryotic environment. Importantly, yeast contains eukaryotic processes with low redundancy and are devoid of immunity mechanisms that counteract T3Es and mask their function. Expression in yeast of effectors from both plant and animal pathogens that perturb conserved cellular processes often resulted in robust phenotypes that were exploited to elucidate effector functions, biochemical properties, and host targets. The genetic tractability of yeast and its amenability for high-throughput functional studies contributed to the success of this system that, in recent years, has been used to study over 100 effectors. Here, we provide a critical view on this body of work and describe advantages and limitations inherent to the use of yeast in T3E research. “Favourite” targets of T3Es in yeast are cytoskeleton components and small GTPases of the Rho family. We describe how mitogen-activated protein kinase (MAPK) signalling, vesicle trafficking, membrane structures, and programmed cell death are also often altered by T3Es in yeast and how this reflects their function in the natural host. We describe how effector structure–function studies and analysis of candidate targeted processes or pathways can be carried out in yeast. We critically analyse technologies that have been used in yeast to assign biochemical functions to T3Es, including transcriptomics and proteomics, as well as suppressor, gain-of-function, or synthetic lethality screens. We also describe how yeast can be used to select for molecules that block T3E function in search of new antibacterial drugs with medical applications. Finally, we provide our opinion on the limitations of *S*. *cerevisiae* as a model system and its most promising future applications.

## Introduction

### Bacterial type III effectors: key interactors with eukaryotic hosts

The type III secretion system is a specialised molecular machinery that directly injects effector proteins inside host cells, constituting the main virulence determinant of many gram-negative bacteria [1]. Type III effectors (T3E) are of special interest in the context of host–pathogen interactions since they target key cellular processes [2,3] and are often multifunctional proteins with a wide array of activities [4]. The number of T3Es with a specific assigned function is still low, especially for those of plant-associated bacteria [5–7]. In addition, although the biochemical activity of several T3Es is already known, their role in infection often remains obscure.

The study of T3Es is particularly complex, as these proteins are usually part of large repertoires that feature internal redundancy and jointly contribute to disease development [8,9]. Thus, in most cases, genetic deletion of an individual T3E has no effect on virulence [10–12]. In addition, overexpression of T3Es is often toxic in the context of the natural host and, as such, hinders monitoring of the intracellular events they trigger [13–15].

### Why choose yeast for a T3E study?

The budding yeast has been used as a model organism for studying eukaryotic processes for more than 50 years [16]. Based on the premise that T3Es target key cellular processes conserved among eukaryotes, heterologous expression in *Saccharomyces cerevisiae* emerged as a promising strategy to investigate their function [17]. Yeast provides powerful genetic, genomic, and proteomic technologies that can be exploited to investigate subcellular localization, biochemical activity, or cellular targets of T3Es [18,19]. For instance, collections of deletion mutants [20] and gene overexpressing strains [21,22], protein chips [23], and synthetic genetic arrays [24] are available. In addition, work with this organism is facilitated by the availability of unique databases and resources comprising genetic and phenotypic information on more than 6,000 functionally annotated genes [25,26]. Finally, yeast is a suitable alternative for gain-of-function analyses of T3E that are naturally delivered into plant cells, since yeast lack the plant immune receptors that recognise effectors—or their virulence activities—and trigger the hypersensitive response (HR), a programmed cell death associated with plant defence [27].

Here, we exhaustively review studies involving the characterisation of T3E function by heterologous expression in *S*. *cerevisiae*. We describe, first, the different methodologies that have been used and, second, the precise biochemical processes altered by T3Es in this heterologous system, highlighting the parallelisms with their function in the original animal or plant context.

## Methodological Approaches for T3E Characterisation in Yeast

A main challenge in the study of T3Es is not only the elucidation of their biological function but also the identification of their physiological targets. As for other proteins, yeast two-hybrid screens have been widely used to determine T3E protein partners inside the host cells [28]. However, toxicity of the effectors has in some cases hindered the use of this methodology (C. Popa, personal communication) [29,30]. Below, we comprehensively describe methodological approaches used so far to characterise T3Es in budding yeast, excluding yeast two-hybrid assays.

### Systems for heterologous expression of T3Es in yeast

The strong *GAL1/10* promoter, which is induced by galactose and repressed by glucose addition, has been used in most studies to drive T3E expression in yeast. Inducibility avoids possible toxicity effects, although a certain level of background expression has been observed [31]. Treatments with varying amounts of repressor and inducer have been used in this system to finely characterise effector activities [32]. To ensure low expression levels of the bacterial effectors, two studies have used the weaker inducible promoters *MET3* or *CUP1* instead [33,34]. This was instrumental to guarantee optimal expression levels of ExoS in a drug inhibitor screen [34]. Four recent studies have employed tetracycline-responsive promoters for controlled expression of the highly toxic *Erwinia* DspA/E, *Ralstonia solanacearum* AWR5, and RipAY or *Pseudomonas aeruginosa* ExoS (C. Popa, personal communication) [35–37]. The main advantage of these systems is the tight regulation of gene expression and the specificity of prokaryotic tet regulators compared to induction by nutrient change that usually causes pleiotropic effects [38].

Strategies to optimise gene expression have also included the use of different copy number vectors. Low-copy centromeric origin plasmids (1–3 copies) have been required for highly sensitive high-throughput screens [39,40] and co-localisation studies, in which low expression allowed distinction of specific structures targeted by the effector [41]. On the other hand, high-copy number plasmids (30–50 copies) using the 2-μ origin of replication have been preferred for T3Es that are not efficiently produced in yeast. In only a few cases, targeted homologous recombination was used to introduce a single effector gene copy and avoid the use of selective media (C. Popa, personal communication) [29,36,42].

### Assays to test effector molecular characteristics and related phenotypes

One of the obvious applications of T3E expression in budding yeast is to test their subcellular location in eukaryotic cells by fusion to fluorescent proteins. Co-localisation studies are simplified by the availability of a comprehensive collection of chromosomally tagged green fluorescent protein (GFP) fusions representing 75% of the proteome, which can be also used for co-immunoprecipitation assays [43].

Inhibition of yeast growth by bacterial effectors is easy to monitor and, thus, has been extensively used as a first step in the search for effector function [39]. For instance, we have identified effectors from three different plant pathogens that inhibited growth in budding yeast (C. Popa, personal communication) [15,40]. Interestingly, yeast growth in the presence of stressors, such as high salt or osmolytes, can increase sensitivity to effectors and has helped in identifying phenotypes caused by effectors that are not apparent under rich-media conditions but are uncovered under different metabolic states.

A number of assays, detailed below, have been used to characterise the precise eukaryotic process affected by T3Es that cause toxicity in yeast. Cell cycle arrest was monitored by flow cytometry analyses or staining the spindle apparatus [42]. Effects on cytoskeleton dynamics and cell polarity [44] have been visualized by using as reporters labelled septin structures and fluorescent markers that associate with actin and actin-related structures [45]. Likewise, specific dyes have been useful to analyse the impact of T3Es on respiration and chitin biosynthesis [45,46]. Kinase assays using specific antibodies or strains with a *lacZ* reporter for mitogen-activated protein kinase (MAPK)-responsive promoters have assisted in the identification of effectors targeting components of MAPK signalling pathways [47,48]. Transcriptional reporters responsive to endoplasmic reticulum (ER) stress and vacuolar and endosomal fluorescent dyes have been successfully exploited to monitor interference with vesicle trafficking and endocytosis [49,50]. The recent adaptation to budding yeast of the carboxypeptidase Y-invertase system to monitor vesicle trafficking [51] holds promise for studies of T3Es modulating this process. Finally, the biological functions of certain T3Es that manipulate Rho GTPase and ADP-ribosyltransferase activities of the host cell have been revealed by measuring bound GTP [33,41] and incorporation of radioactive ADP-ribose [34], respectively.

### Phenotype suppression screens

Collections of deletion mutants allow for assessing functional relationships between nonessential yeast genes and uncovering redundant activities [24]. Based on this, the collection of approximately 5,000 viable yeast deletion mutants has been used to screen for mutants suppressing or enhancing growth inhibition phenotypes caused by T3Es. This approach was termed pathogenic genetic array (PGA) analysis [29], and it was exploited to identify the cellular processes targeted by the effectors IpgB2, OspF, and DspA/E [29,35,52].

Conversely, available yeast overexpressing strain collections [21,22] have been useful in multicopy suppressor screens. This approach relies on the hypothesis that high amounts of effector targets should rescue T3E-triggered phenotypes such as growth inhibition. Remarkably, these screens have identified Rho GTPases as targets of YopT [30], small GTPases as ExoS targets [34], and a MAPK kinase as a suppressor of SteC toxicity [53]. Similarly, using a multicopy suppressor screen in yeast, three distinct genomic loci (two of them encoding small molecular mass GTPases) were identified that suppressed IpaJ activity [54].

### Synthetic lethality screens

Synthetic lethality interactions occur if a mutation in one gene combines with a mutation in another gene to generate a lethal phenotype. Thus, inviable double-mutant progenies can show functional relationships between genes, either by acting in the same pathway or in two parallel pathways that are functionally interconnected [24,55]. Synthetic lethality screens have been successfully utilized to detect T3E-targeted proteins or structures. These screens are based on the assumption that the function of an effector on a cellular target simulates a knockout of that gene, and, thus, expression of the effector in yeast can be regarded as a gene knockout. Functional relationships between yeast genes and the function of a bacterial effector can be inferred by screening for null alleles hypersensitive to its expression. The yeast synthetic lethality interaction network, which lists over 10,000 genetic interactions between 2,795 genes, has been essential for this [55,56]. By this approach, it was found that OspF shares synthetic lethality partners with genes altered in cell wall biogenesis [57]. Data mining of the 83 deletion strains hypersensitive to OspF also identified cell wall biological processes as enriched [52]. More recently, we have optimized synthetic lethality screens by proving that an array of 90 yeast deletion strains covered the majority (69%) of the yeast interactions [49]. The array included nine of 83 deletion strains hypersensitive to OspF [54] that were sufficient to identify 13 genes congruent (sharing synthetic lethality interaction partners) to OspF and involved in cell wall biogenesis-related processes [51]. Screening of this array identified 12 genes congruent to XopE2 and related to the ER stress response [51].

### Proteomic analyses

Interaction studies with T3Es have been hindered by the low concentration of these proteins inside infected host cells. Heterologous expression in yeast oversteps this limitation. In a seminal work, yeast proteins associated with *Escherichia coli* T3Es were isolated by affinity purification and identified by mass spectrometry, providing important clues on their activities [58]. For instance, the EspG effector was found to bind to proteins regulating actin polarization and nuclear division, while the Map effector copurified with a protein controlling vesicle trafficking and a cell wall protein localizing to chitin-rich areas [58].

### Screens for inhibitors of effector-mediated toxicity

Yeast may be used to identify compounds that inhibit T3Es’ activity and restore growth. This chemical biology approach was applied to screen for small molecule inhibitors of *Chlamydia pneumoniae* CopN and *Pseudomonas aeruginosa* ExoS [34,42]. Six potential inhibitors against ExoS toxicity were identified. One of them also reduced the ExoS cytotoxic effect in mammalian cells, and it was shown to act as a competitive inhibitor of ExoS ADP-ribosyltransferase activity in vitro [34]. In the case of CopN, a library of more than 40,000 small molecules was screened for restorers of growth, two of them being identified as specific CopN inhibitors [42].

### Transcriptomic analyses

Transcriptomic analyses in yeast have been exploited in some cases as genome-screening procedures to confirm T3E activities already identified by other techniques. mRNA profiling in yeast cells expressing IpgB2 correlated to those expressing yeast Rho1, proving the proposed IpgB2 GTPase activity [29]. Similarly, yeast transcriptional responses to OspF, which is known to inhibit cell wall integrity MAPK signalling, resulted in downregulation of genes involved in the cell wall integrity pathway [52].

mRNA profiling can also be used as a starting strategy in deciphering conserved processes or molecular targets of T3Es that have unknown functions. The abundance and availability of yeast transcriptomic profiles obtained from mutants in a panoply of environmental conditions enormously facilitate this work [59,60]. In a recent study, we performed microarray and RNA-seq analyses of yeast cells expressing the *Ralstonia solanacearum* AWR5 T3E and found a transcriptomic profile reminiscent of that obtained upon TORC1 inhibition by rapamycin, suggesting that AWR5 targets the TORC1 pathway (C. Popa, personal communication).

## Conserved Cellular Processes Targeted by T3E in Yeast

**Fig 1:**
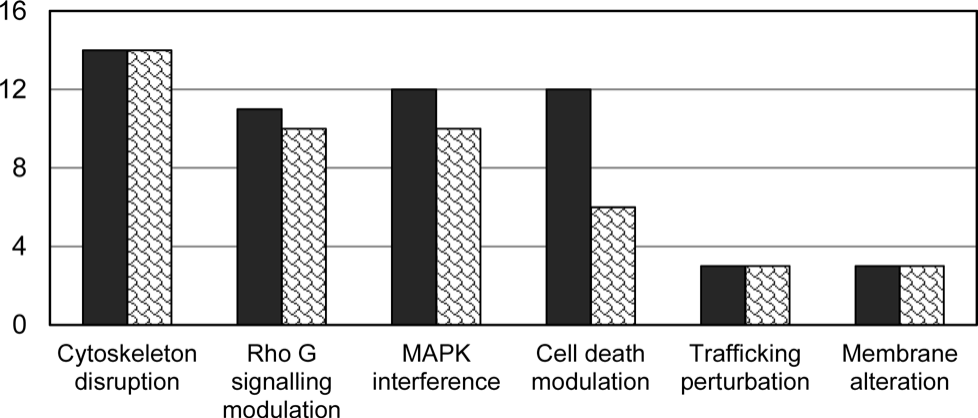
Most common phenotypes observed in *S*. *cerevisiae* upon T3E expression. Black bars indicate total number of effectors described for each phenotype; gray bars, the number also causing growth inhibition.

Heterologous expression of T3Es in *S*. *cerevisiae* has resulted in perturbation of host cellular processes, very often (84% of the cases) accompanied by growth inhibition phenotypes (Fig 1).

Inhibition of yeast growth seems to be highly specific to T3E, as it was only observed for 2% to 8% of randomly selected bacterial genes [34,61]. On the contrary, toxicity is a common output from T3Es when expressed in yeast, as, for instance, 26% of *P*. *syringae* effectors tested inhibited yeast growth [14].

Growth inhibition phenotypes have been exploited to define effector functional domains and biochemical activities. For instance, conserved amino acid residues of YopT, YopE, YopO, YopJ, IpgB2, ExoT, IpaJ, XopE1, and XopE2 are required to cause similar phenotypes in yeast and mammal or plant hosts [15,29,30,33,54,62–64]. Moreover, differential phenotypes in yeast reflected functional differences between homologous T3Es [42,65].

Toxicity in yeast can be caused by alterations in a panoply of cellular activities. To narrow down the precise yeast process targeted by bacterial effectors, a number of assays have been performed, often tracing the cause of growth inhibition to a specific arrest in the cell cycle (Fig 1). In other cases, toxicity is the indirect result of effector interferences with the cytoskeleton, organellar membranes, or specific signalling pathways (see below), so that other observable phenotypes often accompany growth arrest (Table 1).

**Table 1:** List of bacterial T3Es studied in budding yeast. Ref. stands for references. Genera are abbreviated as follows: *S*. *= Salmonella or Shigella*, *X*. *= Xanthomonas*, *E*. *= Erwinia or Escherichia*, *P*. *= Pseudomonas or Pantoea*, *V*. *= Vibrio*, *C*. *= Chlamydia or Citrobacter*.

Effector	Organism	Approaches used in characterisation	Phenotypes in yeast	Ref.
**YopE**	*Yersinia spp*.	GTPase assays, mutational analysis	Growth inhibition, GAP activity	[33]
**YopE and SspA**	*Yersinia spp*.*/ Salmonella typhimurium*	Subcellular localization, flow cytometry, actin staining	Growth inhibition, disruption of actin cytoskeleton	[41]
**YopM**	*Y*. *pestis*	Subcellular localization	Vesicle trafficking-dependent nucleus accumulation	[66]
**YopM**	*Y*. *pestis*	Functional mutational analysis	Identification of critical residues for nuclear targeting	[67]
**YopO/YpkA**	*Yersinia*. *spp*.	Viability assays, actin staining, indirect immunofluorescence	Growth inhibition/cytotoxicity, disruption of actin cytoskeleton	[63]
**YopJ**	*Yersinia*. *spp*.	Phenotype suppression screen, acetylation assays, yeast two-hybrid interactions	Growth arrest, inability to respond to the α factor	[62]
**YspM**	*Y*. *enterocolitica*	Mutational analysis, phenotypic analysis of homologues	Growth inhibition	[65]
**YopE, YopM, YpkA, and SopE2, Sptp, SigD**	*Y*. *enterocolitica and S*. *typhimurium*	CPY-Inv reporter system, Tet-off expression system	Growth inhibition, perturbation of vesicle trafficking	[51]
**SopE2 and SptP**	*S*. *typhimurium*	MAPK phosphorylation assays	Growth inhibition, activation of MAPK signalling	[68]
**SigD**_**R468A**_ **/ SopB**_**R468A**_	*S*. *typhimurium*	Fluorescence staining, functional domain analysis, enzymatic assays	Growth inhibition, actin depolarization	[69]
**SigD**_**R468A**_ **/ SopB**_**R468A**_	*S*. *typhimurium*	Subcellular localization, mutational analysis, indirect immunofluorescence, enzymatic assays, MAPK phosphorylation assays, protein affinity purification	Growth inhibition/growth arrest, disruption of cytoskeleton, inhibition of MAPK signalling, interaction with yeast Cdc42 G12V and Cdc24	[70]
**SteC, SseF**	*S*. *typhimurium*	Screen for effector-triggered phenotypes, fluorescence staining, MAPK phosphorylation assays	Growth inhibition, disruption of actin cytoskeleton	[61]
**SigD**_**R468A**_ **/SopB**_**R468A**_	*S*. *typhimurium*	Fluorescence staining, functional mutational analysis, co-immunoprecipitation, MAPK phosphorylation assays	Growth inhibition, interaction with hCdc42 in yeast	[71]
**SteC**	*S*. *typhimurium*	Phenotype suppression screen, lacZ reporter assays, co-immunoprecipitation assays, fluorescent staining	Growth inhibition, inhibition of MAPK signalling, interaction with GEF Cdc24	[53]
**SspH2**	*S*. *typhimurium*	Cell cycle functional assays	No effect on cell viability or alteration of cell cycle	[72]
**ExoU**	*P*. *aeruginosa*	Fluorescence staining, functional mutational analysis	Growth inhibition /Cytotoxicity	[32]
**ExoU**	*P*. *aeruginosa*	Fluorescence staining, thin-layer chromatography of lipids, enzymatic assays	Growth inhibition/cytotoxicity, membrane alteration, lipase activity	[73]
**ExoT**	*P*. *aeruginosa*	Functional domain analysis	Growth inhibition	[64]
**ExoS**	*P*. *aeruginosa*	Mutational analysis, fluorescence staining, flow cytometry	Growth inhibition, disruption of actin cytoskeleton, inhibition of DNA synthesis	[36]
**ExoS**	*P*. *aeruginosa*	Phenotype suppression screen, screen for inhibitors of effector toxicity, functional mutational analysis, enzymatic assays	Growth inhibition, modulation of Rho G signalling, identification of exosin, a ExoS inhibitor drug	[34]
**VopA**	*V*. *parahaemolyticus*	MAPK phosphorylation assays	Growth inhibition/growth arrest, inhibition of MAPK signalling	[74]
**VopX**	*V*. *cholerae*	Screen for effector-triggered phenotypes	Growth inhibition, MAPK interference	[48]
**EspG**	*E*. *coli/C*. *rodentium*	Actin staining, indirect immunofluorescence	Disruption of microtubule structure	[75]
**EspF, G, H, D, Map**	*E*. *coli*	Fluorescence staining, immunofluorescence, activation of MAPK signalling	Growth inhibition, cell cycle alteration	[45]
EspB, D, F, G, Map and Tir	*E*. *coli*	Protein affinity-purification, μLC-MS/MS	Interaction of EspB, D, F, G, Map and Tir with host proteins	[58]
**34 effectors**	*C*. *trachomatis*	Screen for effector-triggered phenotypes	Growth inhibition	[46]
**CopN**	*C*. *pneumoniae*	Screen for inhibitors of effector toxicity, FACS, fluorescence staining, phenotypic analysis of homologues	Growth inhibition/growth arrest, disruption of microtubule structure, identification of two inhibitor drugs	[42]
**IpgB2**	*S*. *flexneri*	Phenotype suppression screen, mRNA profiling, yeast two hybrid	Growth inhibition, modulation of Rho G signalling	[29]
**OspF**	*S*. *flexneri*	Phenotype suppression screen, MAPK phosphorylation assays, mRNA profiling	Inhibition of MAPK signalling	[52]
**IpaH9.8**	*S*. *flexneri*	Functional domain analysis, enzymatic assays	E3 ubiquitin ligase for MAPKKK	[76]
**IpaJ**	*S*. *flexneri*	Screen for effector-triggered phenotypes	Growth inhibition	[39]
**IpaJ**	*S*. *flexneri*	Phenotype suppression screen	Growth inhibition, identification of IpaJ substrates	[54]
**AvrPtoB**	*P*. *syringae*	Cell death assays	Suppression of stress-induced cell death	[77]
**HopPtoE, HopPtoF, AvrPphE**	*P*. *syringae*	Viability assays	Suppression of Bax-induced PCD	[78]
**27 effectors**	*P*. *syringae*	Screen for effector-triggered phenotypes, cell viability assays, indirect immunofluorescence, domain analysis	Growth inhibition/cell death, loss of respiration	[14]
**HopAA1-1**	*P*. *syringae*	Functional domain analysis	Growth inhibition /cell death	[79]
**29 effectors /HopX1**	*P*. *syringae*	Screen for effector-triggered phenotypes, functional mutational analysis, *lacZ* reporter assays, subcellular localization	Growth inhibition under stress, inhibition of MAPK signalling	[40]
**WtsE**	*P*. *stewartii*	Functional mutational analysis	Growth inhibition	[80]
**21 effectors**	*X*. *euvesicatoria*	Screen for effector-triggered phenotypes, functional mutational analysis	Growth inhibition/growth arrest & cell death, growth inhibition under stress	[15]
**XopE2**	*X*. *euvesicatoria*	Synthetic lethality screen, *lacZ* reporter assays	Perturbation of vesicle trafficking	[49]
**DspA/E**	*E*. *amylovora*	Fluorescent staining, cell viability assays	Growth inhibition, disruption of actin cytoskeleton, perturbation of trafficking	[50]
**DspA/E**	*E*. *amylovora*	Phenotype suppression screen, actin staining, HPLC, cell labelling and thin-layer chromatography, phosphorylation assays	Disruption of cytoskeleton, down-regulation of sphingolipid pathway	[35]

### Disruption of the cytoskeleton

**Fig 2:**
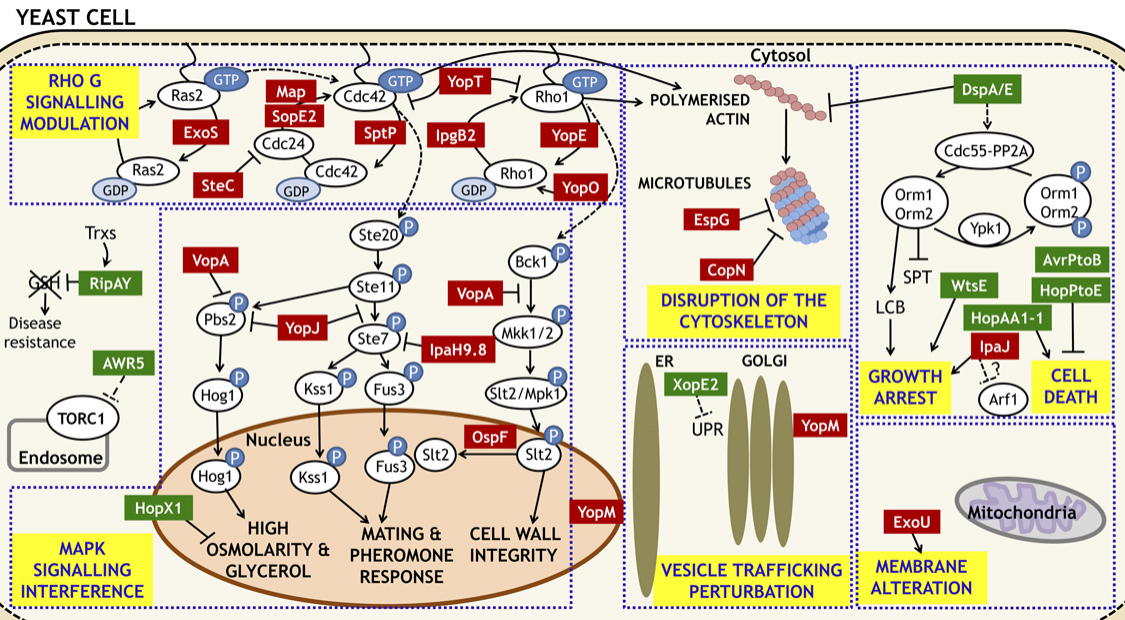
Yeast cellular processes targeted by bacterial T3Es. The key processes targeted are marked in yellow and their components separated by dotted areas. Plant-associated effectors (green squares) and animal-associated effectors (red squares) are indicated next to the activities they modulate. Arrows indicate activation and T symbols indicate inhibition. Abbreviations: UPR: unfolded protein response; SPT: serine palmitoyltransferase; LCB: long chain bases; GSH: glutathione; Trxs: thioredoxins; P: phosphate group. Other common abbreviations are given in the text.

Disruption and/or rearrangement of the host cell cytoskeleton are the most common strategies that bacterial T3Es employ to facilitate infection [81,82]. Precisely, 25% of the characterised effectors target cytoskeletal structures (Figs 1 and 2). For instance, DspA/E caused defects in cell polarization and endocytosis, two processes that depend on a functional actin cytoskeleton [50]. The effect on actin is often mediated by direct targeting of Rho-GTPases through the guanine nucleotide exchange factor activity of T3Es that share a WxxxE motif (as further discussed below), such as EspD and Map [8]. On the contrary, DspA/E seems to act through downregulation of sphingolipid biosynthesis, which hinders proper localization of actin regulators [50].

Other T3Es target the microtubule cytoskeleton, preventing normal functioning of the cell cycle [83]. It was shown that CopN-expressing cells stalled at the G2/M transition and showed abnormal microtubule spindle formation in both mammals and yeast [42]. Other T3Es, such as ExoT or PheA, use the same strategy to redirect resources in the host cell to promote bacterial multiplication [84,85]. Similarly, EspG was shown to disrupt microtubule structures and perturb actin polarization in both mammals and yeast [45,75]. The double effect of EspG on actin and microtubules is likely mediated by modulation of G protein signalling (see below) [86].

### Rho family of small GTPases: “favourite” targets of bacterial effector proteins

Rho GTPases constitute the protein family most frequently targeted by T3Es in yeast (Table 1). In fact, 11 effectors representing 20% of the T3E with a described target in yeast were found to specifically manipulate these eukaryotic proteins. Interference with Rho GTPases has been shown to facilitate bacterial invasion or to help bacteria escape phagocytosis and immune responses triggered in their animal and plant hosts [87–89]. Rho-family GTPases function as molecular switches regulating membrane trafficking, actin dynamics, cell cycles, or nuclear import in response to extracellular stimuli [90]. These small G proteins are activated to a GTP-bound state by guanine nucleotide exchange factors (GEFs) and inactivated by cytoplasmic GTPase activating proteins (GAPs) to a GDP-bound state [91]. Bacterial T3Es modulate Rho signalling by mimicking GAPs and GEFs, inhibiting or activating, respectively, small G protein signalling events (Fig 2).

YopE was shown to cause loss of actin cytoskeletal polarity in yeast, blocking bud formation and actin rings [41], in agreement with its GAP activity in mammalian cells [33]. However, other bacterial T3Es, such as IpgB2, Map, SopE2, and SifA function as GEFs, activating Rho GTPases both in yeast and mammalian systems [29,68], likely in order to promote lesion formation [92]. Interestingly, effectors from plant-associated bacteria, such as WtsE and AvrE, bear a WxxxE motif shared by GEF-acting T3Es [8] and require this conserved motif to promote disease [93]. Bacterial infection may involve sequential secretion of effectors with GEF and GAP activities. In *Salmonella*, the GEF-like SopE2 effector is required in the initial stages of infection for actin rearrangement and bacterial invasion, while the antagonizing GAP-like SptP is injected later to allow cell recovery and completion of bacterial internalization [94–96]. In a different scenario, the effector YpkA/YopO functions by mimicking guanine nucleotide-dissociation inhibitors, which sequester inactive Rho GTPases into the cytosol and inhibit their signalling without altering the GDP/GTP exchange [97].

Besides Rho-GTPases, the activity of the other two GTPase subfamilies, Cdc42 and Rac, is also modulated by T3Es [98]. For instance, SopE2 appears to alter the activity of both Rho1 and Cdc42 GTPases in yeast, stimulating signalling through both the filamentation and mating pathway and the cell wall integrity pathway [68]. Targeting of multiple kinase pathways by T3Es has also been observed in their natural context: YopE was shown to target RhoA, Rac, and Cdc42 in human cells [33]. *S*. *cerevisiae* has been used to gain insight into the specificity of T3Es toward the different GTPases. IpgB2 was shown to function as a GEF for Rho1, while SopB_R468A_/SigD_R468A_ interacted with yeast or human Cdc42 but not Rac1 when coexpressed in *S*. *cerevisiae* [29,70,71]. Finally, SteC was shown to exert an indirect effect on GTPases by binding to the Cdc42 GEF from both yeast and human [53].

### Inhibition of MAP kinase signalling

Inhibition of MAPK phosphorylation is also a common effect of the interaction of T3Es with the eukaryotic cell signalling machinery (Table 1 and Fig 2). Effectors from both animal and plant pathogens were shown to inhibit specific steps in the four best-characterized MAPK signalling pathways in yeast [99], which have conserved components and cell surface receptors shared among all eukaryotes [100].

Expression of HopX1 in yeast showed that it attenuated the activation of the high osmolarity/glycerol (HOG) MAPK pathway under stress conditions, without alteration of expression or nuclear dynamics of the Hog1 MAPK [40]. Yeast growth inhibition caused by this effector and modulation of HOG signalling were dependent on its intact transglutaminase activity. Interestingly, this activity is also essential for virulence as well as recognition by cellular defence in plants [101]. It can be speculated that interaction with and modification of a plant MAPK signalling component would drive HopX1 recognition in resistant hosts and may contribute to virulence in susceptible hosts. Along the same line, *Shigella flexneri* OspF was shown to inhibit MAPKs of the cell wall integrity, the HOG, the pheromone response, and the filamentous growth pathways [52]. Importantly, HopX1 expression in yeast provided the first clues to its activity, similarly to OspF, for which simultaneously independent research identified its enzymatic activity.

Other bacterial effectors operate directly on components of MAPK cascades. For instance, IpaH9.8 acts as an E3 ubiquitin ligase causing proteasomal degradation of the MAP kinase kinase (MAPKK) Ste7, reducing the phosphorylation level of its MAPK targets and inhibiting the yeast pheromone response/mating MAPK pathway [76]. Likewise, YopJ inhibits both the yeast pheromone response and HOG MAPK pathways by preventing the activation of the equivalent MAPKK (Fig 1) [62]. Interestingly, the YopJ-like effector VopA inhibits the HOG MAPK pathway and the cell wall integrity pathway, by preventing phosphorylation of MAPKs Hog1 and Mpk1, respectively [74]. However, unlike YopJ, VopA caused growth arrest in yeast, indicating that the level at which the T3E exerts its inhibition is key for the final outcome. Subsequent discoveries revealed that YopJ inhibits MAPKK by acetylation of two residues in its activation loop while VopA acetylates both these two residues and a lysine required for ATP binding, which explains the incongruent phenotypes observed in yeast. Lastly, VopX was proposed to interact with components of the yeast cell wall integrity MAPK pathway [48].

### Modulation of pathogen-triggered cell death

In plants, localized cell death at the site of infection is a typical defence outcome triggered by the specific recognition of bacterial T3Es by the plant immune system, and it is referred to as the hypersensitive response (HR). The HR is typically associated with plant disease resistance and elicitation of additional defence responses. Consequently, several effectors from phytopathogenic bacteria have been shown to function as inhibitors of programmed cell death. Screening for *P*. *syringae* HR suppressors identified four T3Es, including AvrPtoB, able to suppress Bax-induced cell death in both plants and yeast, indicating that their targets are likely conserved across kingdoms [77,78]. In a similar screen, five *Xanthomonas* effectors suppressed plant cell death, mediated by overexpression of components of immunity-associated MAP kinase cascades [102]. Among them, AvrBs1 was also able to suppress activation of the HOG MAPK pathway, suggesting that the target of this effector is conserved in eukaryotic organisms [103]. On the other hand, by virtue of their virulence activity, certain T3Es may trigger cell death when overexpressed in plants and yeast. For example, we found that XopX, AvrRxo1, XopB, XopE1, and XopF2 from *X*. *euvesicatoria* caused cell death or chlorosis when expressed in *Nicotiana benthamiana* and/or tomato plants [15]. In yeast, XopB and XopF2 attenuated cell proliferation, while AvrRXo1, XopX, and XopE1 were cytotoxic. Interestingly, cytotoxicity of XopX and AvrRXo1 was associated with cell-cycle arrest at G0/1 [15]. Similarly, *P*. *syringae* HopAA1-1 leads to cell death in both yeast and plants [14]. Exploitation of the yeast model system, where effector function can be studied in the absence of immune receptor proteins and the HR response, has been very useful and has identified effectors that caused different phenotypes in yeast and plants. As an example, the tyrosine phosphatase HopAO1 and the cysteine protease HopN1 were shown to arrest yeast growth only if their enzymatic activities were intact [14]. Interestingly, the same activities were also required to inhibit HR cell death in plants [103–105]. This suggests that biochemical functions are conserved in different cell contexts, although this may have disparate physiological consequences [14].

### Alteration of membrane structure or vesicle trafficking

Certain bacterial effectors induce host cell permeation and membrane damage to ensure pathogen entry and survival inside host cells [106]. For example, ExoU from *P*. *aeruginosa* encodes a phospholipase A2, whose activity releases free fatty acids from membrane phospholipids causing membrane damage to different organelles, fragmentation of the vacuole in yeast [73], and acute cytotoxicity in mammals [107].

In addition to manipulating the cytoskeleton and endomembrane system, intracellular pathogenic bacteria have learned to escape from phagosomal degradation by modulating host–vesicle trafficking. Effects of DspA/E from *E*. *amylovora* and EspG from enteropathogenic *E*. *coli* on this pathway was described to be a consequence of their above-mentioned activities altering the cytoskeleton or G protein signalling, respectively [50,108,109]. Other effectors were shown to target trafficking from the ER to the nucleus. We found that *Xanthomonas* XopE2 perturbed this molecular signalling by interfering with the activation of the unfolded protein response (UPR) upon ER stress induction [49]. T3E manipulation of the UPR may help the pathogen to escape from phagocytic pathways and cell death. ExoS also was recently shown to alter intracellular trafficking through ADP-ribosylation of similar mammal and yeast targets [34,110]. Interestingly, the ADP-ribosylation activity is encoded in a C-terminal domain essential for yeast growth inhibition in both ExoS and ExoT, which also have a Rho-GTPase activity domain (see above) [36,64].

In other cases, bacterial effectors exploit vesicular transport to their benefit. For instance, proper nuclear localization of YopM from *Yersinia pestis* in both yeast and mammalian cells requires a functional vesicle trafficking [66,67]. Yeast has been a robust model to study YopM trafficking dynamics and to identify its leucine-rich repeat (LRR) domain as necessary for nuclear translocation.

## Concluding Remarks

In this work, we present a comprehensive overview on latest studies using *S*. *cerevisiae* systems biology to uncover T3E function. We have shown how yeast offers unique and versatile resources for the study of effector proteins from many pathogenic bacteria. Growth inhibition phenotypes are common amongst T3Es and can be exploited in both small-scale and genome-wide functional analysis. Further characterisation in yeast has expedited the parallel work performed with the host cells, in which T3Es are naturally injected and common targets have often been identified in the two systems. We have cited many examples where yeast was instrumental in identifying the physiological targets of effector proteins. In some cases, such as IpaH9.8, the yeast target was different from the relevant mammalian target, but it allowed classification of an entire family of effector proteins as novel E3 ubiquitin ligases [76].

However heterologous, characterisation of T3Es in yeast has many limitations. First, proper expression of the bacterial gene or the posttranslational modifications acquired in the native animal or plant cells may not be achieved in yeast. This may be an important reason why the majority of T3Es show no apparent phenotype when expressed in yeast. Against this hypothesis are examples like ExoU, which requires binding to ubiquitin or ubiquitin-modified proteins and is functional in both animal and yeast cells, which still contain the required ubiquitylated proteins [111,112]. Second, the specific process affected by the T3E may not be sufficiently conserved across kingdoms, for example, because budding yeast lacks key proteins in a pathway, or their structure and/or function is not sufficiently conserved to be targeted by T3Es. For instance, regulation of growth and cell cycle progression in yeast depends on cell-size checkpoints, whereas mammalian cells respond to extracellular growth factors [113]. The fact that effectors, such as YopE, often recognise their targets in natural host cells better than in yeast is suggestive of this [33]. An additional limitation derives from the fact that bacterial effectors may target physiological processes that are specific to the host, such as the plant and mammalian innate immune response. For example, to avoid recognition and activation of defence responses, certain T3Es of phytopathogenic bacteria manipulate the function of components of the plant immune system that are not conserved in yeast. Therefore, the elucidation of physiological functions of T3Es that target such components of plant immunity may not be possible in yeast. Still, in some circumstances, phenotypes in yeast can only be uncovered by the activation of specific signalling pathways, thereby allowing the investigator insight into the molecular mechanism targeted by the T3SS effector. Thus, yeast remains an effective, fast, and robust means to identify cellular pathways affected by T3Es and will continue contributing to the understanding of bacterial pathogenesis.
